# Hepatitis B Virus Genotype Study in West Africa Reveals an Expanding Clade of Subgenotype A4

**DOI:** 10.3390/microorganisms9030623

**Published:** 2021-03-17

**Authors:** Rayana Maryse Toyé, Damien Cohen, Flor Helene Pujol, Amina Sow-Sall, Gora Lô, Kunikazu Hoshino, Masashi Mizokami, Fabien Zoulim, Maud Lemoine, Coumba Touré-Kane, Isabelle Chemin

**Affiliations:** 1 Institut National de la Santé et de la Recherche Médicale (Inserm) U1052, CRCL, 151 Cours Albert Thomas, 69003 Lyon, France; damien.cohen@inserm.fr (D.C.); fabien.zoulim@inserm.fr (F.Z.); 2 Institut de Recherche en Santé, de Surveillance Epidémiologique et de Formation (Iressef), BP 7325, Diamniadio 20000, Senegal; aminassow@gmail.com (A.S.-S.); goralo808@yahoo.fr (G.L.); ctourekane@yahoo.co.uk (C.T.-K.); 3 Laboratorio de Virología Molecular, CMBC, Instituto Venezolano de Investigaciones Científicas (IVIC), Caracas 1020A, Venezuela; fhpujol@gmail.com; 4 Department of Infectious, Respiratory, and Digestive Medicine, Graduate School of Medicine, University of the Ryukyus, Okinawa 903-0215, Japan; kuhoshino-ryk@umin.ac.jp; 5 Research Institute, Genome Medical Sciences Project, National Center for Global Health and Medicine, Ichikawa, Chiba 272-8516, Japan; mmizokami@hospk.ncgm.go.jp; 6 Division of Digestive Diseases, St Mary’s Hospital, Imperial College London, London SW7 2AZ, UK; m.lemoine@imperial.ac.uk

**Keywords:** Hepatitis B virus, genotype, subgenotype, Africa, classification, sequence

## Abstract

Hepatitis B virus (HBV) classification comprises up to 10 genotypes with specific geographical distribution worldwide, further subdivided into 40 subgenotypes, which have different impacts on liver disease outcome. Though extensively studied, the classification of subgenotype A sequences remains ambiguous. This study aimed to characterize HBV isolates from West African patients and propose a more advanced classification of subgenotype A. Fourteen HBV full-length genome sequences isolated from patients from The Gambia and Senegal were obtained and phylogenetically analyzed. Phylogenetic analysis of HBV genotype A sequences isolated from Senegalese and Gambian patients exhibited separate clusters from the other known and confirmed subgenotypes A (A1, A2, A6). Most of the sequences (10/14) clustered with an isolate from Cuba, reported as subgenotype A4 (supported by maximal bootstrap value). Four isolates from The Gambia and Senegal clustered separately from all other subgenotypes and samples sequenced in the study. Three of which from The Gambia, designated as an expanding clade of subgenotype A4, exhibited a mean inter-subgenotypic nucleotide divergence over the entire genome sequence higher than 4% in comparison with the other subgenotypes and the other isolates sequenced in the study, except with subgenotype A4 isolates (3.9%), and this was supported by a maximal bootstrap value. The last one from Senegal seemed to be an expanding subgenotype close to the new clade of A4. Amino acid analysis unveiled a novel motif specific to these isolates. This study revealed an expanding evolution of HBV subgenotype A and novel amino acid motifs. It also highlighted the need for a consensus regarding the analysis and classification of HBV sequences.

## 1. Introduction

Hepatitis B virus (HBV) isolates were initially classified into nine genotypes (A-I) and a putative genotype (J) based on intergenomic sequence divergence in the complete genome of at least 8% [1,2,3,4]. As new HBV genotypes were identified, they were gradually classified into subgenotypes based on a divergence of 4–8% [4]. This classification is of the utmost importance as it may contribute to tracing the spread of HBV in the world [5], and, as more HBV isolates are sequenced, especially from more remote regions, the number of subgenotypes may progressively expand [6].

HBV genotypes and subgenotypes have distinct geographical and clinical associations [7]. HBV genotypes display distinct geographical and ethnic distribution. For example, genotypes B and C are prevalent in Asia, while genotypes A and D are prevalent in Europe, the United States, and Central Africa. Genotype E is by far the dominant genotype in West Africa (exclusively found in Africans) and has a very low intra-genotypic diversity suggesting that this genotype has spread only recently. No subgenotypes have been reported for HBV genotype E, although separate lineages have been described [8]. There is still a number of debates on the classification of HBV subgenotypes. This is the case, for instance, for subgenotype A, widely distributed across the globe, with A1 and A2 being historically called Ae for European and Aa for African and Asian [9].

Between 1997 and early 2020 [5,9,10,11,12,13,14,15,16,17,18], HBV genotype A was subdivided into eight subgenotypes, though only three of these (A1, A2, and A6) seem to be clearly defined to date. Indeed, subgenotypes A3, A4, A5, A7, and A8 have been reported in Africa, but their identification is not based on genomic-length sequences or strong phylogenetic analysis according to agreed definitions, leading to bias [19] and debate regarding their further reclassification into quasi-subgenotype A3 (QA3) [20,21].

Current HBV classification is based on agreed definitions of nucleotide sequence diversity [6,22] with a threshold of >7.5% for genotypes, between 4–7.5% for subgenotypes, and between 1–3% for clades. This latter classification also depends on monophyletic clustering and strong bootstrap support. However, the bootstrap value considered significant to support these discriminations is unclear. In 2010, Pourkarim et al. suggested considering a value of at least 75% to delineate a new subgenotype [20], while more recently, McNaughton et al. chose a bootstrap threshold of 70% [23].

The need to set up clear and consensual guidelines for subgenotyping, given the increasing amount of HBV sequencing data, has already been highlighted [13,23]. In their meticulous analysis, McNaughton presented a comprehensive set of HBV reference sequences at the genotype and subgenotype level and defined clear guidelines for sequence analysis and classification [23].

This study aimed to characterize HBV sequences from West African patients according to the most recent published guidelines.

## 2. Materials and Methods

We investigated samples from patients enrolled in the Prolifica (Prevention of liver fibrosis and cancer in Africa) program from The Gambia [24] and Senegal. The Gambia Government/Medical Research Council (MRC) Joint Ethics Committee (SCC 1266 West African Treatment Cohort for Hepatitis B) validated this study. The complete HBV genome was amplified by nested PCR using High fidelity Platinum™ Taq DNA polymerase (Invitrogen™, Thermo Fisher Scientific, Waltham, MA, USA). The amplicons were enzymatically purified and sequenced using sets of primers as previously described [25,26]. Primer/DNA mixes were sent to the Genewiz Sanger sequencing service (Genewiz, Leipzig). The resulting sequences were assembled and edited using the SeqMan™ software (DNASTAR, Inc., Madison, WI, USA) and, after visual inspection, the consensus sequences were used for analyses. All of the sequences generated and used in the study, as well as the reference sequences, were submitted to the jumping profile Hidden Markov Model (jpHMM) tool [27] to detect possible recombination events. Nucleotide sequence data have been deposited in the GenBank database under the accession numbers MW567967-MW567980.

We generated 2 datasets, one comprising our sequences and reference sequences of all HBV genotypes (A–J), to classify our sequences within HBV genotypes, and another comprising our sequences and reference sequences of HBV genotype A, to classify our sequences exclusively within HBV genotype/subgenotype A. As suggested by McNaughton and colleagues [23], we performed alignment using the multiple alignment fast Fourier transform (MAFFT) tool [28]. For each dataset, we performed a model test, and a phylogenetic tree was constructed based on the maximum likelihood method (Bootstrap 1000 replicates) using the Molecular Evolutionary Genetics Analysis version 7 (MEGA 7) software [29]. We used the aLRT (approximate likelihood-ratio test for branches) method to generate another tree using the PhyML (Phylogeny.fr) platform [30] to assess the branch support for the second dataset. We added the proposed reference sequences (completed with missing A7 and A8) retrieved from the GenBank database for all different HBV genotypes and subgenotypes. One subgenotype A7 sequence of the 10 available and 1 subgenotype A8 of the 2 available were selected. The resulting tree was visualized using MEGA 7 software. Sequences were then submitted to the Basic Local Alignment Search Tool (BLAST) to find the most similar sequences [31]. Pairwise association analysis was performed on the second dataset using the (MEGA 7) software [29].

Surface gene (preS1/preS2 and S domains) fragments from whole-genome sequences were translated into amino acids using the Expasy web tool and aligned using MAFFT. Amino acid analysis in the surface gene was manually performed at key positions [32].

## 3. Results

We generated 14 HBV genomic-length sequences for genotype A originating from 7 participants from The Gambia and 7 from Senegal. Recombination analysis showed that HBV genotype A sequences used in the study did not display any recombination event, except one isolate recently proposed as novel subgenotype A8 (MN585097) [18], which contained an uncertainty region, i.e., a region in the sequence where the posterior probability of the predicted subtype is lower than a certain threshold. The model test showed that the best substitution model with the lowest BIC (Bayesian information criterion) score was the general time-reversible using a discrete Gamma distribution with five rate categories and assuming that a certain fraction of sites were evolutionarily invariable (GTR + G + I).

**Figure 1:**
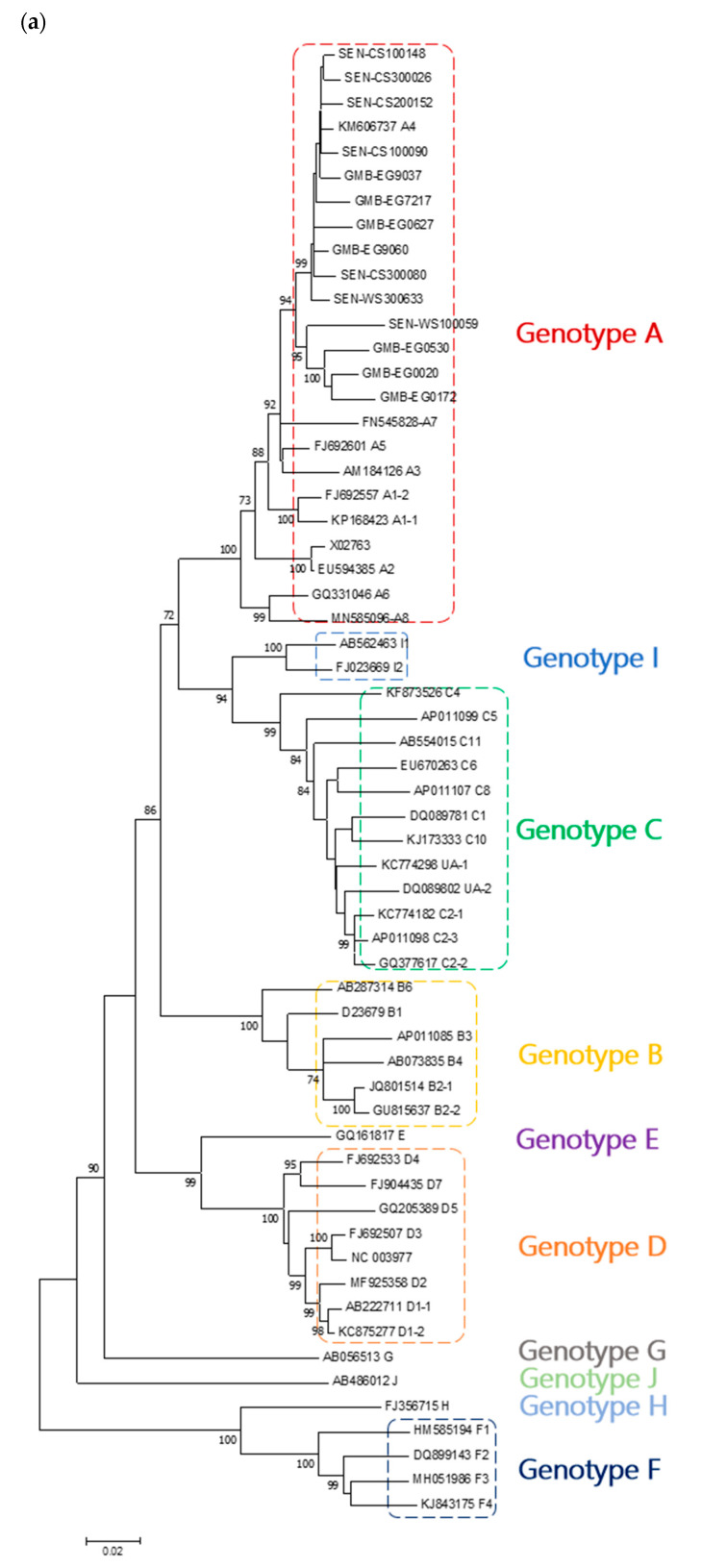

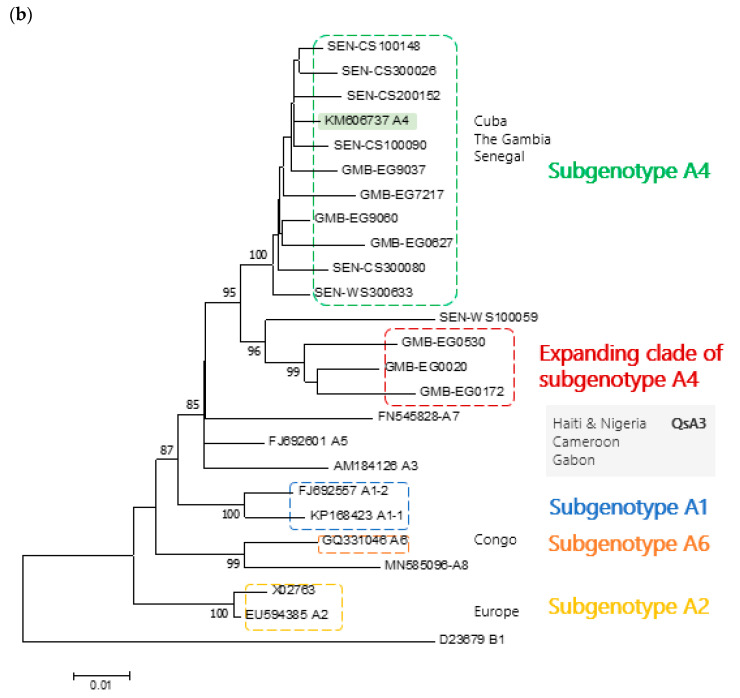
Genomic length maximum likelihood phylogenetic tree constructed based on Hepatitis B virus (HBV) sequences isolated in patients from The Gambia and Senegal and (**a**) genotype A–J reference sequences and (**b**) genotype/subgenotype A reference sequences retrieved from the GenBank database and rooted on an HBV genotype B sequence. Samples sequenced in this study are identified by the prefixes GMB (The Gambia) and SEN (Senegal). The numbers at each node correspond to the bootstrap values (greater than 70%) obtained with 1000 replicates.

**Figure 2:**
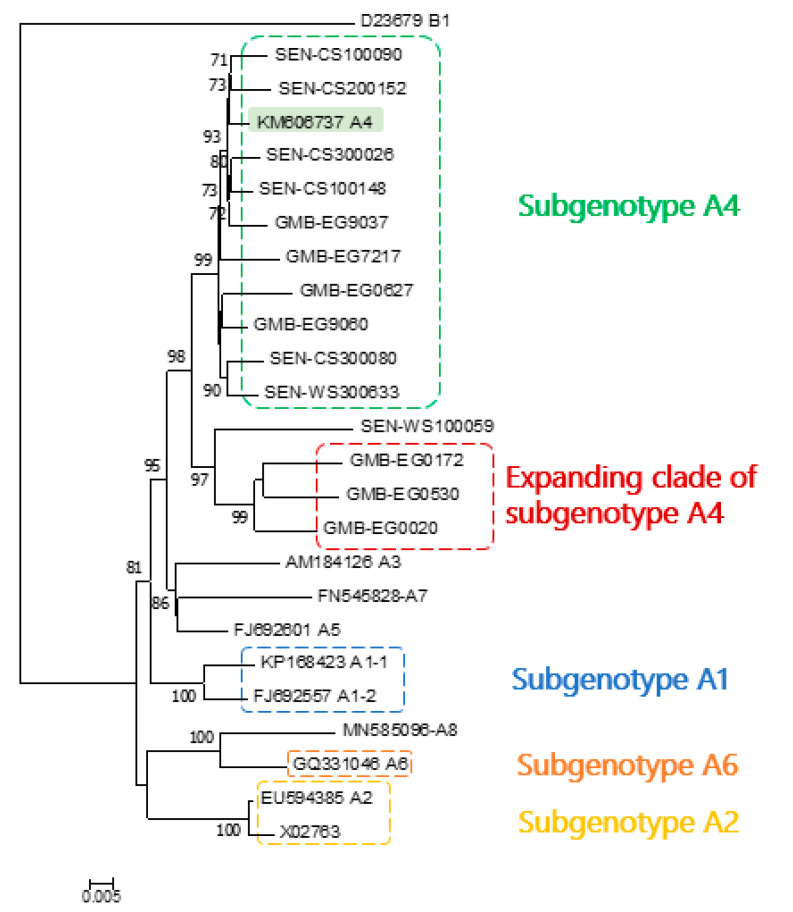
Phylogenetic tree reconstructed using the maximum likelihood method implemented in the PhyML program (v3.1/3.0 approximate likelihood-ratio (aLRT)). The general time-reversible (GTR) substitution model was selected, assuming an estimated proportion of invariant sites (of 0.480) and five gamma-distributed rate categories to account for rate heterogeneity across sites. The gamma shape parameter was estimated directly from the data (gamma = 0.504). Reliability for the internal branch was assessed using the aLRT test (SH-Like). Samples sequenced in this study are identified by the prefixes GMB and SEN. The numbers at each node correspond to the aLRT supports (greater than 70%).

Three phylogenetic trees were generated using the datasets generated; the first comprised our 14 sequences and 46 HBV reference sequences of all genotypes, leading to a total number of 60 sequences; the second comprised our 14 sequences, 10 HBV genotype A reference sequences (one from each subgenotype except for A1 and A2 which had 2 reference sequences) and one HBV genotype B used as a root, leading to a total number of 25 sequences; the third comprising the same sequences as in the second dataset but using the aLRT branch support method. Figure 1a shows that all sequences from The Gambia and Senegal belong to genotype A. Indeed, these sequences clustered close to HBV genotype A reference sequences with a maximal bootstrap value. Figure 1b shows that these sequences, except four, clustered close to an isolate from Cuba (KM606737) reported as subgenotype A4 [8]. Indeed, these sequences genetically differed from subgenotype A1, A2, A3, A5, A6, A7, and A8 with a maximal bootstrap value and were distinct from isolates previously reported in this African region. The remaining four sequences formed a separate cluster from all other known sequences supported by a very strong bootstrap value (96%). In addition, three of these sequences originating from The Gambia formed a distinct clade strongly supported by high bootstrap value (99%) and proposed an expanding clade of subgenotype A4. Figure 2 shows that there is a basic agreement between aLRT supports and bootstrap supports shown in Figure 1b. BLAST analysis performed on all sequences also revealed that the Cuban isolate A4 was one of the most similar sequences.

As shown in Table 1, the divergence between our expanding clade of subgenotype A4 sequences and subgenotypes A1 to A8 was between 3.9 and 6.4%, but the divergence between the 10 other samples sequenced in the study and the A4 isolate from Cuba was lower than 3% (1.7 ± 0.004; data not shown). The intragenomic divergence within this expanding clade of subgenotype A4 was 2.9%.

As shown in Table 2, amino acid analysis in the S gene identified the subgenotype-specific *ayw1* motif in nine of our sequences. A new motif, ayw1/2, due to the presence of a Glycine replacing an Alanine at position 159 (G159A), was identified in three of the five remaining sequences, forming the expanding clade of subgenotype A4.

## 4. Discussion

Our study aimed at characterizing HBV isolates in patients from The Gambia and Senegal. We presented, in this study, the first entire genome sequences of HBV from Senegalese patients. In our search for the optimal method of analysis for our sequences, we unveiled some inconsistencies that can lead to confusion despite previous attempts to set up clear guidelines. We performed multiple sequence alignment using MAFFT and tree construction using the maximum likelihood method with 1000 bootstrap resampling. In most studies, ClustalW was the method used for multiple sequence alignment [5,9,13,16]. In addition, the neighbor-joining method and the Kimura two-parameter (K2P) or 6-parameter model were the most reported for phylogenetic tree construction [5,8,9,11,13,16]. Moreover, the reliability of the clusters was assessed by bootstrapping with 1000 replicates in the majority of studies, although the significance of a strong bootstrap value was not clear and seemed to be subjective. Although some authors proposed a threshold value of 75% for subgenotyping, a recent study conducted in the same team reported a new HBV subgenotype D based on a bootstrap value of 55% [18,20]. We believe this value may be too low to meet the subgenotype definition. Moreover, the authors introduced a novel subgenotype A8 based on two sequences supported by a strong bootstrap and nucleotide divergence greater than 4%. However, the recombination analysis showed that one of the proposed A8 sequences presented an uncertainty region.

In a recent study, McNaughton and coworkers carried out a meticulous analysis of HBV sequences and suggested the use of MAFFT for alignment and the maximum likelihood method for phylogenetic analysis and considered a bootstrap value > 70% as significant [23]. However, we noticed that the authors did not mention subgenotype A7 and A8 sequences. The absence of the A7 sequence may be due to the fact that it is assimilated into quasi-subgenotype A3. This explanation would only make sense if the authors had not considered A4 and A5 that are also believed to be part of QA3. Regarding the A8 subgenotype, its discovery was reported after the authors published their own work.

Along with the bootstrap method, we generated a phylogenetic tree using the aLRT method to assess the branch support better. The method is fast and may be more accurate for assessing the reliability of the clusters.

All sequences from The Gambia and Senegal clustered in the same group with high bootstrap support. In particular, our sequences were distinct from isolates that were previously reported from this region in Africa. Interestingly, most of our sequences clustered with an isolate from Cuba (KM606737) reported as subgenotype A4, supported by BLAST analysis. A similar observation has been recently reported in Senegalese children though the analysis was based on the partial preS1/preS2/S region [33]. In addition, three of the four remaining sequences exhibited a separate cluster from all other sequences supported by a very strong bootstrap value, which led us to propose an expanding clade of HBV subgenotypeA4. These findings suggest an ongoing evolution of subgenotype A sequences in West Africa.

As previous studies suggested [6,19,22], the genetic distance between samples and reference sequences should be between 4–7.5% to classify new sequences as a novel subgenotype. We calculated genetic distances between our samples and other subgenotypes A sequences using isolates belonging to subgenotypes A1, A2, A3, A4, A5, A6, A7, and A8 as reference sequences. Genetic distances between three of our sequences and subgenotypes A1 to A8 were greater than 4%, except for subgenotype A4 (3.9%). We, therefore, suggest that these isolates might be an expanding clade of subgenotype A4. However, the high intragenomic divergence within the expanding clade of subgenotype A4 (2.9%) suggests that it may have been endemic in this region for a long time. The low mean intragenomic divergence between 10 of our sequences and the subgenotype A4 from Cuba (1.7%) suggests that this subgenotype may have a recent story of endemicity in West Africa.

We identified a new amino acid motif, ayw1/2, in the three sequences forming the expanding clade of subgenotype A4 and one sequence very close to these. However, one sequence outside this group exhibited the same motif. It will be interesting to explore these findings on a larger number of samples from the West African region.

Our sequences clustered with the subgenotype A4 from Cuba. As previously observed by Loureiro et al., the A4 isolate from Cuba clustered with an A4 isolate from The Gambia [8]. Thus, to investigate the relationship between our sampling location and Cuba, we retrieved HBV sequences from Cuba and performed phylogenetic analyses (data not shown). However, no additional isolate was found close to our samples. It might be difficult to determine the relationship between our samples and the isolate from Cuba. However, the similarity of our African HBV sequences to a sequence from Cuba [8], where this sequence was an exception, could be linked to the movement of medical doctors from Cuba to The Gambia in recent decades.

## 5. Conclusions

Our study shows that a general agreement is needed for HBV classification into subgenotypes. Based on recent analysis guidelines, we reported the presence of subgenotype A4 in West African patients and an expanding clade of subgenotype A4, which might be a novel subgenotype A of HBV in this region. A larger number of sequences would undoubtedly allow the further description of the landscape of HBV subgenotypes A in this area. Finally, we strongly recommend the standardization of HBV classification and dialog between experts in the field to avoid discrepancies and confusion in the future.

## Figures and Tables

**Table 1:** Percentage (mean ± SD) of nucleotide divergence between Hepatitis B virus (HBV) subgenotype A full sequences and expanding clade of subgenotype A4.

Subgenotype	Expanding Clade A4
A1	5.3 ± 0.004
A2	5.4 ± 0.003
A3	5.0 ± 0.004
A4	3.9 ± 0.004
A5	4.2 ± 0.004
A6	5.5 ± 0.004
A7	5.6 ± 0.006
A8	6.4 ± 0.004
Expanding clade A4	(2.9 ± 0.002)

**Table 2:** Identification of subgenotype specific amino acid motifs in the S gene.

				Amino Acid Position in the S Surface Gene					
	**Sample**	**Genotype**	**Serotype**	**122**	**160**	**127**	**159**	**140**	**or 134**
**1**	CS100090	A4	ayw1	R	K	P	A	T	F
**2**	CS100148	A4	ayw1	R	K	P	A	T	F
**3**	CS200152	A4	ayw1	R	K	P	A	T	F
**4**	CS300026	A4	ayw1	R	K	P	A	T	I
**5**	**CS300080**	A4	**ayw1/2**	R	K	P	**G**	T	F
**6**	**EG0020**	**New clade A4**	**ayw1/2**	R	K	P	**G**	T	F
**7**	**EG0172**	**New clade A4**	**ayw1/2**	R	K	P	**G**	T	F
**8**	**EG0530**	**New clade A4**	**ayw1/2**	R	K	P	**G**	T	F
**9**	EG0627	A4	ayw1	R	K	P	A	T	F
**10**	EG7217	A4	ayw1	R	K	P	A	T	F
**11**	EG9037	A4	ayw1	R	K	P	A	T	F
**12**	EG9060	A4	ayw1	R	K	P	A	T	F
**13**	**WS100059**	Close to **clade A4**	**ayw1/2**	R	K	P	**G**	**T**	**F**
**14**	WS300633	A4	ayw1	R	K	P	A	T	F

## Data Availability

The data presented in this study are openly available in GenBank, accession numbers MW567967-MW567980].
